# Surface Expressed Nucleolin Is Constantly Induced in Tumor Cells to Mediate Calcium-Dependent Ligand Internalization

**DOI:** 10.1371/journal.pone.0015787

**Published:** 2010-12-23

**Authors:** Ara G. Hovanessian, Calaiselvy Soundaramourty, Diala El Khoury, Isabelle Nondier, Josette Svab, Bernard Krust

**Affiliations:** CNRS-Université Paris Descartes, Unité Régulation de la Transcription de Maladies Génétique, Paris, France; University of Helsinki, Finland

## Abstract

**Background:**

Nucleolin is one of the major proteins of the nucleolus, but it is also expressed on the cell surface where is serves as a binding protein for variety of ligands implicated in tumorigenesis and angiogenesis. Emerging evidence suggests that the cell-surface expressed nucleolin is a strategic target for an effective and nontoxic cancer therapy.

**Methodology/Principal Findings:**

By monitoring the expression of nucleolin mRNA, and by measuring the level of nucleolin protein recovered from the surface and nucleus of cells, here we show that the presence of nucleolin at the cell surface is dependent on the constant induction of nucleolin mRNA. Indeed, inhibitors of RNA transcription or translation block expression of surface nucleolin while no apparent effect is observed on the level of nucleolin in the nucleus. The estimated half-life of surface nucleolin is less than one hour, whereas that of nuclear nucleolin is more than 8 hours. Nucleolin mRNA induction is reduced markedly in normal fibroblasts that reach confluence, while it occurs continuously even in post-confluent epithelial tumor cells consistent with their capacity to proliferate without contact inhibition. Interestingly, cold and heat shock induce nucleolin mRNA concomitantly to enhanced mRNA expression of the heat shock protein 70, thus suggesting that surface nucleolin induction also occurs in response to an environmental insult. At the cell surface, one of the main functions of nucleolin is to shuttle specific extracellular ligands by an active transport mechanism, which we show here to be calcium dependent.

**Conclusion/Significance:**

Our results demonstrate that the expression of surface nucleolin is an early metabolic event coupled with tumor cell proliferation and stress response. The fact that surface nucleolin is constantly and abundantly expressed on the surface of tumor cells, makes them a preferential target for the inhibitory action of anticancer agents that target surface nucleolin.

## Introduction

Nucleolin is a multifunctional RNA- and protein-binding protein ubiquitously expressed in exponentially growing eukaryotic cells. It is involved in fundamental aspects of transcription, cell proliferation and growth [1], [2]. Nucleolin is found at several locations in cells: in the nucleolus it controls many aspects of DNA and RNA metabolism [3]; in the cytoplasm it shuttles proteins into the nucleus and provides a post-transcriptional regulation of strategic mRNAs [4], [5]; and on the cell surface where it serves as an attachment protein for several ligands from growth factors to microorganisms [6], [7], [8], [9], [10], [11], [12]. Surface and cytoplasmic nucleolin are differentiated from nuclear nucleolin by a slight shift in their isoelectric point, probably as a consequence of specific co- and/or post-translational modifications that determine routing of the newly synthesized nucleolin to the nucleus or the plasma membrane [6]. Moreover, surface and cytoplasmic nucleolin appear to be down regulated independently of nuclear nucleolin, since under some experimental conditions marked reduction of surface/cytoplasmic nucleolin occurs without any apparent effect on the level or nucleolar localization of nuclear nucleolin [13]. Both nuclear and surface nucleolin are phosphorylated; nuclear nucleolin is phosphorylated by casein kinase 2 (CKII) at interphase and by cell-cycle control CDC2 kinase during mitosis, whereas surface nucleolin is phosphorylated by casein kinase-like ecto-protein kinase [1], [2].

In spite of the fact that nucleolin does not have a hydrophobic transmembrane domain for anchorage in the plasma membrane, cross-linking of surface nucleolin with a specific monoclonal antibody results in its clustering in the plasma membrane. This clustering occurs at the external side of the plasma membrane and is dependent on its association with the intracellular actin cytoskeleton [6]. An actin based motor protein, the nonmuscle myosin heavy chain 9, could serve as a physical linker between surface nucleolin and actin [14]. Upon stimulation of cell proliferation, cytoplasmic nucleolin is translocated via small vesicles to the surface through a temperature-dependent but unconventional secretory pathway, since such an active translocation is not affected by inhibitors of the classical pathway for secretion through the endoplasmic reticulum and Golgi apparatus [6]. Interestingly, N-linked glycosylation of cytoplasmic nucleolin appears to be an essential requirement for the expression of nucleolin on the surface of different types of cells [15]. Surface nucleolin serves as a low affinity receptor for HIV-1 and various growth factors that interact with its C-terminal domain containing nine repeats of the tripeptide arginine-glycine-glycine, known as the RGG or GAR domain [10], [16], [17], [18], [19]. Binding of these ligands results in clustering of cell-surface nucleolin in lipid raft membrane microdomains before endocytosis of the ligand-nucleolin complex [10], [17], [19]. Accordingly, surface nucleolin could shuttle ligands between the cell surface and the nucleus thus act as a mediator for the extracellular regulation of nuclear events [18], [20], [21].

Interestingly, most of ligands of surface nucleolin play a critical role in tumorigenesis and angiogenesis. For example, among surface nucleolin binding growth factors and proteins, midkine and pleiotrophin can transform cells, whereas on endothelial cells they exert both mitogenic and angiogenic effect [22], [23]. Urokinase that also binds the RGG domain of nucleolin is implicated in mechanisms regulating pericellular proteolysis, cell-surface adhesion, and mitogenesis [21], [24]. Other surface nucleolin binding proteins such as laminin-1, factor J, L- and P-selectins, and hepatocyte growth factor that are involved in tumor development, induce cell differentiation, regulate cell adhesion, leukocyte trafficking, inflammation, and angiogenesis [25], [26], [27], [28], [29], [30]. The tumor homing peptide F3 that binds both endothelial and tumor cells is internalized via surface nucleolin, while endostatin that inhibits angiogenesis binds nucleolin on the surface of endothelial cells before translocation to the nucleus [31], [32]. Consequently, the functional blockade or down regulation of surface nucleolin in endothelial cells inhibits migration of endothelial cells and prevents capillary-tubule formation [13], [14].

Following interaction with various ligands, surface nucleolin might be implicated directly or indirectly in signal transduction events [33]. For example, surface nucleolin has been shown to serve the binding partner for receptor protein tyrosine phosphatase-sigma ectodomain in skeletal muscle [34], whereas the interaction of surface nucleolin with ErbB receptor tyrosine kinases induces receptor dimerization, phosphorylation and to anchorage independent growth [35]. The binding of P-selectin to human colon carcinoma cells is shown to induce tyrosine phosphorylation of surface nucleolin and formation of a signaling complex containing nucleolin, phosphatidylinositol 3-kinase (PI3-K) and p38 MAPK [29]. Finally, ligand binding to surface nucleolin could generate high transitory intracellular Ca^2+^ membrane fluxes involving SOC-like channels, and thus initiate signaling events [15]. Consistent with this, plasma membrane localized nucleolin with nucleophosmin interact with K-Ras and play a critical role in signal transduction via the MAPK pathway [36].

These various reports on the implication of surface nucleolin in growth of tumor cells and angiogenesis highlight surface nucleolin as a promising target for cancer therapy. Accordingly, we recently reported that the progression of established human breast tumor cell xenografts in the nude mice is suppressed by targeting surface nucleolin with a specific antagonist, the HB-19 pseudopeptide, that binds the RGG domain of nucleolin [13], [16]. HB-19 prevents binding of growth factors to cells, triggers calcium entry into cells, inhibits MAP kinase activation, and down regulates surface nucleolin without affecting nuclear nucleolin [7], [9], [13], [15], [16], [18], [19]. In a more relevant tumor model, we showed that HB-19 treatment for several months delays significantly the onset and frequency of spontaneous melanoma in RET mice, and reduces visceral metastatic nodules while displaying no toxicity to normal tissue [37], [38]. Studies on the melanoma-derived tumor cells demonstrated that HB-19 treatment could restore contact inhibition, impair anchorage-independent growth, and inhibit expression of specific genes implicated in tumorigenesis [37]. Previously, guanosine-rich quadruplex-forming oligodeoxynucleotides (GROs) that interact with surface nucleolin and/or intracellular nucleolin have been shown to be promising agents for treatment of cancer [39], [40], [41]. The aptamer AS1411 is the most recent GRO that is currently being tested in Phase II clinical trials. The proposed model for AS1411 mechanism of action involves inhibition of molecular interactions between nucleolin and its binding partners, such as NF-κB essential modulator (NEMO also known as IKKγ), arginine methyltransferase 5 (RPMT5) and the instability AU-rich element of bcl-2 mRNA [42], [43], [44].

In this study, we provide evidence to show for the first time that surface nucleolin is a *de novo* synthesized protein constantly induced in tumor and endothelial cells. Accordingly, inhibitors of RNA transcription or translation block the expression of surface nucleolin in the absence of any apparent effect on nuclear nucleolin. The estimated half-life of surface nucleolin is about 45 minutes whereas that of nuclear nucleolin is more than 8 hours. At the cell surface, nucleolin mediates internalization of specific ligands in a calcium-dependent manner. Consequently, chelating divalent calcium from the culture medium blocks ligand and surface nucleolin internalization. Finally, the observation that the level of nucleolin mRNA is reduced by inhibition of mRNA transcription but not by mRNA translation is consistent with an induction mechanism that is a primary induction process not requiring the synthesis of other proteins. Taken together, our results demonstrate that the induction and down regulation of surface nucleolin is associated with the proliferative capacity of cells, and occur independently of nuclear nucleolin. This latter could account, at least in part, for the lack of toxicity observed during the prolonged treatment of tumor bearing mice with HB-19 [13], [37]. The differential expression and specific functions of surface and nuclear nucleolin indicate the need to distinguish between these two forms of nucleolin in future studies in order to avoid possible misinterpretations of experimental data.

## Results

Surface nucleolin can be isolated conveniently by the capacity of HB-19 to form a stable and an irreversible complex with it [9]. Consequently, incubation of cells with biotinylated HB-19 and followed by purification of cell extracts using avidin-agarose provide an efficient method for monitoring surface nucleolin expression. In different types of cells, HB-19 binds surface nucleolin in a dose-dependent manner with maximum binding occurring at 1–5 µM concentration [12], [13], [15], [45]. In this study, measuring the level of surface nucleolin separately of nuclear nucleolin was the key step to demonstrate that surface nucleolin is a continuously induced protein in different types of tumor cells.

### Surface nucleolin is constantly induced in cells

**Figure 1:**
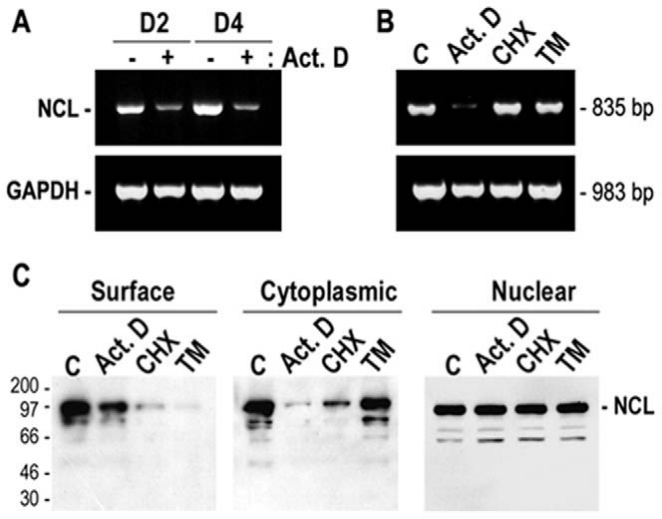
Induction of surface nucleolin expression in the human breast cancer cell line, MDA-MB-231. The expression of nucleolin (NCL) and GAPDH mRNA (Panels A and B: by RT-PCR) and nucleolin protein (Panel C: by immunoblotting of cell extracts) were monitored in MDA-MB-231 cells cultured in the absence (lane - or C) or in the presence of 5 µg/ml actinomycin D (lanes Act. D) or 50 µg/ml cycloheximide (lanes CHX) or 10 µg/ml tunicamycin (lanes TM) for 5 hours. A. Constitutive induction of nucleolin mRNA in proliferating MDA-MB-231 cells without (lanes -) or with (lanes +) actinomycin D treatment at days 2 and 4 after passage, respectively (lanes D2 and lanes D4). B. Expression of nucleolin mRNA level in serum-stimulated subconfluent cells in the absence (lane C) or presence of actinomycin D, cycloheximide, and tunicamycin. C. Nucleolin expression at the cell surface (panel Surface), in cytoplasmic (containing both surface and cytoplasmic proteins) and nuclear extracts. MDA-MB-231 cells cultured for 5 hours with various inhibitors were further incubated (20°C, 45 min) with 5 µM HB-19/Btn in order to monitor the cell surface expressed nucleolin. Samples of the purified surface nucleolin (material from 5×10^6^ cells) and crude cytoplasmic and nuclear extracts (material from 2×10^6^ cells) were analyzed by immunoblotting using anti-nucleolin monoclonal antibody D3. The size of RT-PCR product for nucleolin and GAPDH was 835 and 983 bp, respectively. Note. The stained PAGE-SDS gel showed no apparent differences in cytoplasmic extracts that were obtained from cells treated with various inhibitors compared to the control sample.

Our previous studies by laser scanning confocal immunofluorescence microscopy have suggested that transcriptional and translational events are implicated for the expression of nucleolin at the cell surface [6]. In view of this, we investigated the expression of nucleolin mRNA in MDA-MB-231 cells along with the level of nucleolin protein at the cell surface, in the nucleus and in the nuclear-free subcellular fraction that contains both cytoplasmic and plasma membrane proteins (referred to as cytoplasmic fraction). We first showed that nucleolin mRNA is constantly induced in subconfluent and confluent MDA-MB-231 cells, since inhibiting transcription with actinomycin D decreases nucleolin mRNA by more than 85% (Fig. 1A). We next investigated the expression of nucleolin mRNA and protein in confluent cells in response to inhibitors of transcription, translation, and N-glycosylation (Fig. 1B and 1C). Consistent with the results in Fig. 1A, actinomycin D prevented markedly nucleolin mRNA expression, while the protein synthesis inhibitor cycloheximide had no apparent effect, thus suggesting that nucleolin mRNA induction is a direct induction process not requiring newly synthesized proteins. Furthermore, the induction of nucleolin mRNA is a specific event since it occurs in the absence of any apparent effect on the relative amounts of the house-keeping GAPDH mRNA (Fig. 1A and 1B).

As for the nucleolin protein expression, the reduced levels of nucleolin mRNA in actinomycin D treated cells is accompanied by marked reduction of surface and cytoplasmic but not nuclear nucleolin, thus indicating that the expression of surface/cytoplasmic nucleolin is dependent on the constant induction of nucleolin mRNA. Consistent with this, translation of the induced nucleolin mRNA is necessary for the expression of both cytoplasmic and surface nucleolin, since cycloheximide treatment of cells causes a specific decline of cytoplasmic/surface nucleolin without an apparent effect on nucleolin mRNA (Fig. 1B and 1C). We have recently reported that N-linked glycosylation is an essential requirement for the expression of nucleolin at the cell surface [15]. Interestingly, N-glycosylation inhibitor tunicamycin has no apparent effect on the induction of nucleolin mRNA, which is translated to generate cytoplasmic but not surface nucleolin (Fig. 1B and 1C). Therefore nucleolin is synthesized in the presence of tunicamycin but its translocation to the cell surface is prevented because it is not glycosylated. It should be noted that the level of nucleolin in the nucleus remains constant in cells treated with actinomycin D, cycloheximide or tunicamycin that cause a marked reduction of surface nucleolin, thus indicating that these inhibitors exert a specific effect on the expression of surface nucleolin (Fig. 1C). Accordingly, confocal laser immunofluorescence microscopy studies have indicated that nuclear nucleolin does not translocate to the cytoplasm when surface nucleolin levels are reduced in the presence of the transcription and translation inhibitors [6]. Nevertheless, It is noteworthy to mention that in the presence of actinomycin D or cycloheximide, nucleolin becomes re-distributed from the nucleolus into the nucleoplasm (Data not shown).

**Figure 2:**
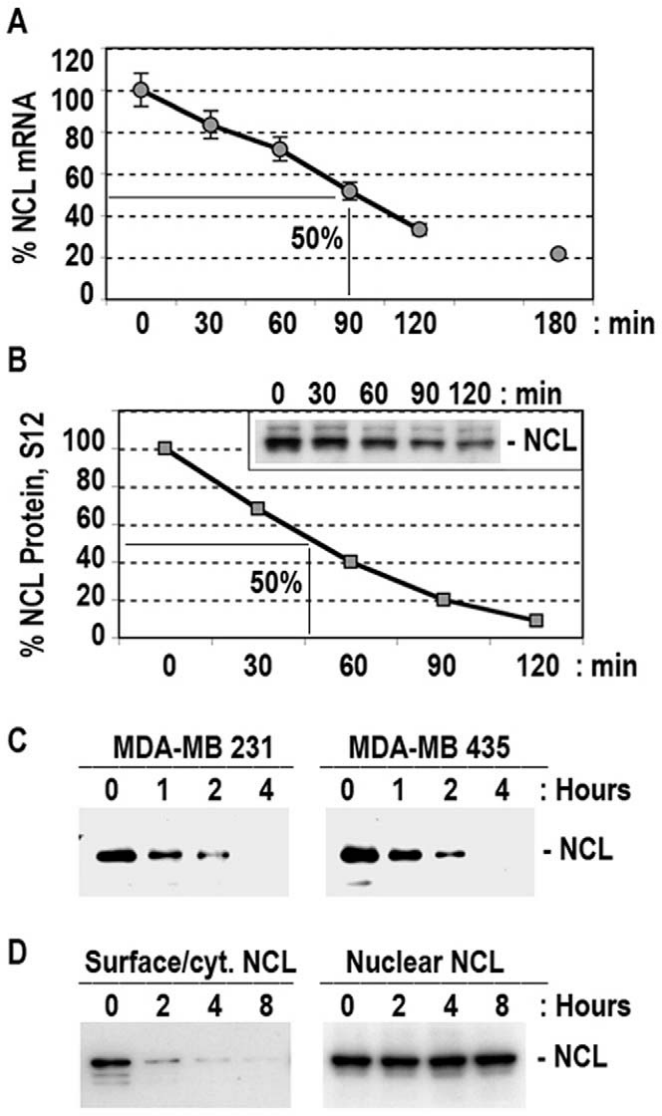
Half-life of nucleolin mRNA and the cytoplasmic and surface nucleolin. A. Half life nucleolin mRNA. MDA-MB-231 cells in the presence of 5 µg/ml actinomycin were cultured for 30, 60, 120 and 180 min before extraction of cells. Time 0 represents the control sample. Nucleolin mRNA expression was analyzed by Q-PCR using GAPDH RNA as endogenous control RNA (Materials and methods). B. Half-life of cytoplasmic/surface nucleolin protein. MDA-MB-231 cells were cultured in the presence of 50 µg/ml of cycloheximide for 30, 60, 90 and 120 min. Nucleolin protein was monitored by immunoblotting of crude cytoplasmic cell extracts. A section of the gel is shown. The intensity of nucleolin protein bands was quantified by using the NIH image software. C. Half-life of the cell-surface expressed nucleolin. MDA-MB-231 and MDA-MB-435 cells were cultured for 1, 2 and 4 hours. At time 0 or 1, 2 and 4 hours of treatment with cycloheximide (50 µg/ml), cells were transferred to room temperature for the recovery of surface nucleolin using HB-19/Btn. Sections of the immunoblot at the position of nucleolin are presented. D. Nucleolar nucleolin is a highly stable protein compared to its cytoplasmic/surface counterpart. MDA-MB-435 cells cultured in the presence of cycloheximide for 0, 2, 4, and 8 hours were analyzed by immunoblotting for nucleolin expression in crude cytoplasmic and nuclear extracts containing cytoplasmic/surface (panel Surface/Cyt. NCL) and nuclear (panel Nuclear NCL) nucleolin.

The half-life of nucleolin mRNA monitored by RT-PCR (not shown) or by RT-Q-PCR (Fig. 2A) is estimated to be about 90 minutes. The half-life of the nucleolin protein monitored by immunoblotting of crude nuclear-free subcellular fractions and the purified surface nucleolin is estimated to be about 45 minutes and less than one hour, respectively (Fig. 2B and 2C). On the other hand, no apparent effect is observed on the level of nuclear nucleolin in cycloheximide treated cells for 8 hours, when there is almost complete disappearance of cytoplasmic/surface nucleolin (Figure 2D). These observations suggest that surface/cytoplasmic nucleolin represents the majority of the newly synthesized protein detectable at a given time, the proportion of which in different types of cells is less than 10% of nucleolin located in the nucleus [6], [46]. Consequently, our results do not exclude the potential translocation of some of the newly synthesized nucleolin to the nucleus, since a slight increase would not modify significantly the overall level of nuclear nucleolin. Whatever is the case, the absence of an apparent effect on nuclear nucleolin after 8 hours of cycloheximide treatment indicates clearly that nuclear nucleolin has a prolonged half-life. In accord with this, other workers have reported that a drop or rise of nucleolin mRNA have no apparent effect on the level of nucleolin protein as measured in total cell extracts [47]. Therefore, the differential expression of cytoplasmic/surface nucleolin in respect to nuclear nucleolin is characterized by constant induction and the short half-life of cytoplasmic/surface nucleolin.

**Figure 3:**
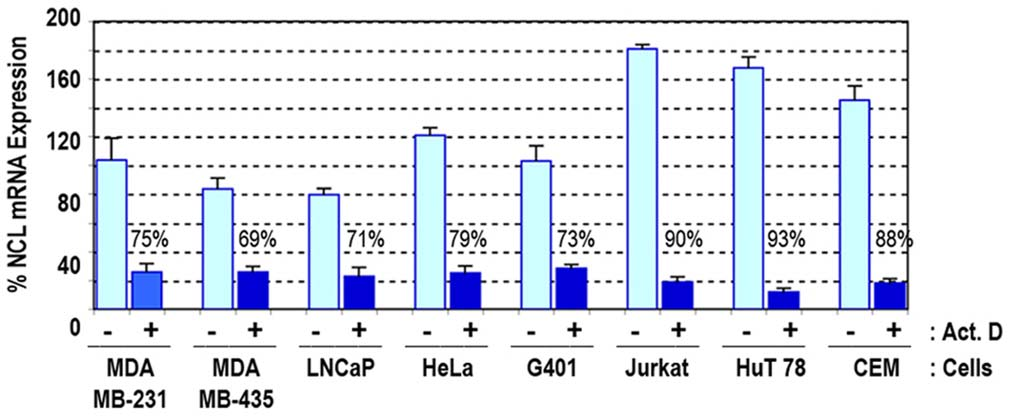
Relative expression of nucleolin mRNA in various human cell lines. Subconfluent adherent cells (MDA-MB-231, MDA-MB-435, LNCaP, HeLa, G401) and cells in suspension at 5×10^5^ cells/ml (Jurkat, HuT 78, CEM) were cultured in the absence or presence of 5 µg/ml actinomycin. After 4 hours, extracts were prepared and analyzed for nucleolin mRNA by Q-PCR using 28S ribosomal RNA as endogenous control RNA as described in the Materials and methods. For comparative purposes, the level of nucleolin expression in the absence of actinomycin D in MDA-MB-321 cells was considered 100%. The percent inhibition of nucleolin mRNA expression in the presence of actinomycin D is given individually for each cell type.

Consistent with the results observed in the human breast carcinoma cells (MDA-MB-231, MDA-MB-435), the constant induction of nucleolin mRNA is also observed in the human carcinoma (LNCaP, HeLa, G401) and leukemia (Jurkat, HuT 78, CEM) cell lines that were investigated. In these cells, the steady state levels of nucleolin mRNA were reduced by 69–93% in response to treatment with actinomycin D during a period of four hours (Fig. 3). Among the different types of cancer cells, the expression of nucleolin mRNA is higher in leukemia compared to the epithelial tumor cells, which is in accord with a recent report showing that nucleolin is over expressed in acute myelogenous leukemia cells [48].

### Down regulation of surface nucleolin in HB-19 treated cells does not affect induction of nucleolin mRNA

**Figure 4:**
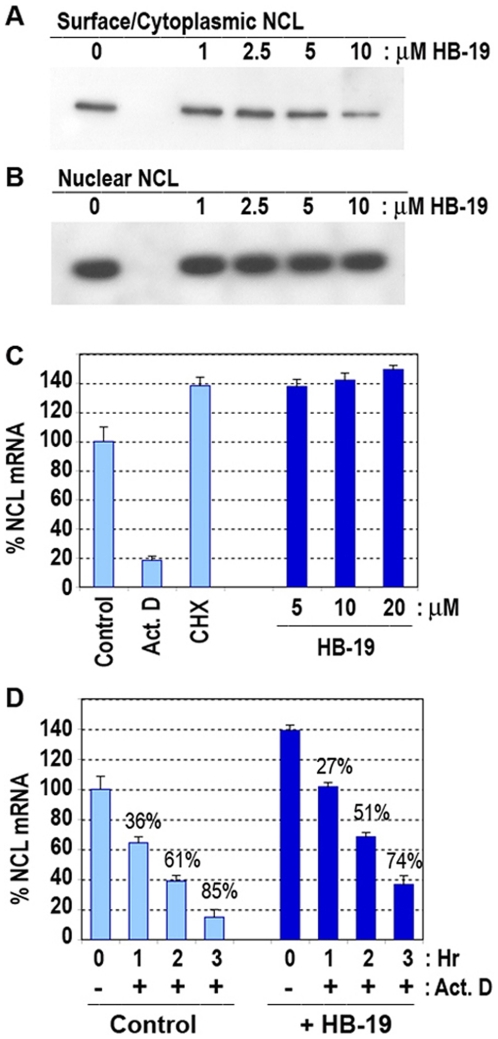
HB-19 down regulates expression of surface nucleolin without affecting the induction of nucleolin mRNA. A–B. Specific reduction of surface but not nuclear nucleolin in HB-19 treated cells. MDA-MB-231 cells were cultured with HB-19 at 0, 1, 2.5, 5 and 10 µM of for 5 hours before preparation of cytoplasmic (i.e, containing cytoplasmic and plasma membrane proteins) and nuclear extracts. Material from 2×10^6^ cells was analyzed by immunoblotting for the detection of nucleolin. Sections at the position of nucleolin are presented. PAGE-SDS gels containing similar samples were stained with Brilliant Blue G-Colloidal Concentrate in order to monitor the protein profile in the crude cell extracts (not presented). C. Nucleolin mRNA level is not affected in HB-19 treated cells. MDA-MB-231 cells in the absence (control) or presence of actinomycin D (5 µg/ml), cycloheximide (50 µg/ml), and HB-19 (5, 10, 20 µM) were cultured for 5 hours before analysis of nucleolin mRNA expression by Q-PCR using GAPDH RNA as endogenous control RNA. The expression of nucleolin mRNA in the control sample was considered as 100%. D. The half-life of nucleolin mRNA in HB-19 treated cells is delayed slightly compared to untreated cells. MDA-MB-231 cells in the absence (control) or presence of 10 µM HB-19 were cultured for 24 hours before addition of actinomycin D (5 µg/ml). After 1, 2, and 3 hours, nucleolin mRNA expression was carried out by Q-PCR using GAPDH RNA as endogenous control mRNA. The percent inhibition of nucleolin mRNA expression given above the histograms was calculated in comparison with the control or HB-19 treated sample at 0 hour.

Treatment of cells with HB-19, the nucleolin antagonist-pseudopeptide, results in a dose dependent reduction of surface/cytoplasmic nucleolin without any apparent effect on nuclear nucleolin (Fig. 4A–B), or on the expression of other cytoplasmic proteins (data not shown). Therefore, the marked drop of cytoplasmic/surface nucleolin in HB-19 treated cells is a specific and a selective effect. In spite of this, no apparent effect is observed on the level of nucleolin RNA in HB-19 treated cells (Fig. 4C), even after 24–48 hours of treatment when cytoplasmic nucleolin becomes no longer detectable [13]. Consistently, the steady state levels of nucleolin mRNA was found to be slightly higher in HB-19 treated compared to untreated cells; an effect that might reflect enhanced stability of nucleolin mRNA as a consequence of the translational block, as it is the case with the translational inhibitor cycloheximide (Fig. 4C, histogram CHX). Accordingly, the estimated half-life of nucleolin mRNA in HB-19 treated cells, as monitored in the presence of actinomycin D, is slightly higher compared to untreated cells (Fig. 4D).

### The expression of nucleolin on the surface of endothelial cells is due to the induction of nucleolin mRNA

**Figure 5:**
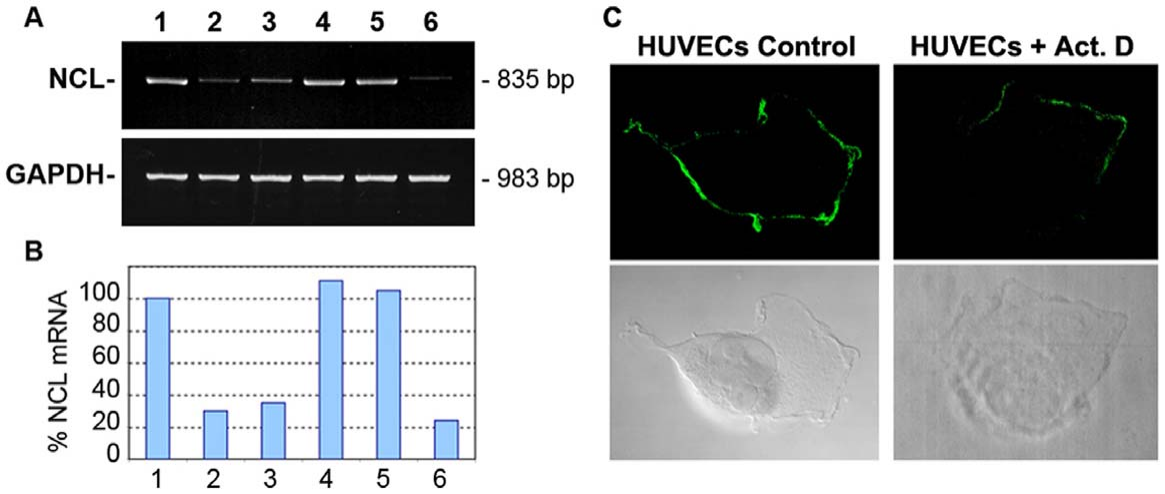
Induction of nucleolin mRNA in human umbilical vein endothelial cells (HUVECs). A. Nucleolin mRNA levels were monitored by RT-PCR in HUVECs cultured in the EBM-2 medium containing 2% FBS in the absence (lane 1) or presence of 5 µg/ml actinomycin D for 6 hours (lane 2). Serum starved cells for 24 hours (lane 3) were stimulated for 6 hours by suspending in the EBM-2/2% FBS medium (lane 4) or in fresh starvation medium containing 100 ng/ml VEGF in the absence (lane 5) or presence of actinomycin D (lane 6). The size of RT-PCR product for nucleolin and GAPDH was 835 and 983 bp, respectively. B. The nucleolin PCR-bands were quantified by using the NIH image software. The histograms represent the intensity of the PCR products in respect to the control sample that was considered 100%. C. Reduced expression of surface nucleolin in HUVECs in response to inhibition of RNA transcription. HUVECs cultured on glass slides were treated or not with actinomycin D (5 µg/ml) for 6 hours. Cells were then washed in PBS, incubated (45 min, 20°C) with anti-nucleolin mAb D3 to result clustering of surface nucleolin, fixed with PFA, and processed for laser confocal immunofluorescence microscopy [6]. The scans of cells toward the middle cell layer are presented with the respective phase contrasts.

The expression of nucleolin on the surface of human umbilical vein endothelial cells (HUVECs) was recently suggested to be the consequence of translocation of nucleolar nucleolin to the surface when HUVECs are activated by vascular endothelial growth factor (VEGF) [14]. Like other cell types however, we show here that surface nucleolin expression is associated with induction of nucleolin mRNA in activated HUVECs. Indeed, expression of nucleolin on the surface of HUVECs as well as nucleolin mRNA are markedly reduced in cultures incubated with actinomycin D for few hours (Fig. 5A–B, lanes 1–2; Fig. 5C). Nucleolin mRNA is also reduced in HUVECs starved for 24 hours, but addition of complete culture medium rapidly restores nucleolin mRNA levels (Fig. 5A–B, lanes 3–4). Interestingly, addition of VEGF is also sufficient to induce nucleolin mRNA levels in starved HUVECs (Fig. 5, lanes 5–6), thus indicating that induction of nucleolin mRNA is one of the early events in endothelial cells in response to activation by VEGF.

### The constant induction of nucleolin mRNA is associated with the proliferative capacity of tumor cells

**Figure 6:**
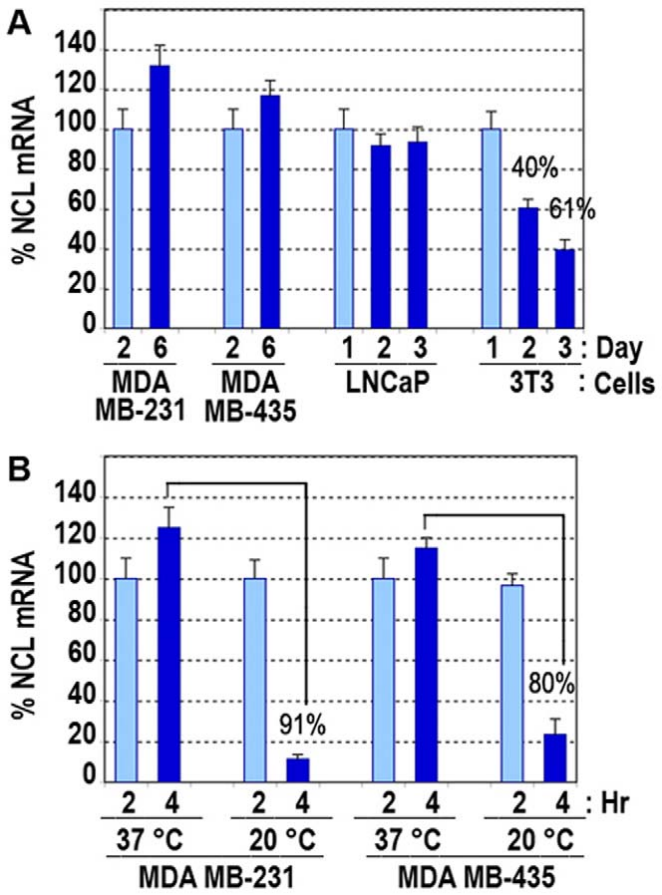
The constant induction of nucleolin mRNA is associated with the proliferative capacity of tumor cells. A. Nucleolin mRNA is induced constantly in post-confluent epithelial tumor cells compared to normal fibroblasts. The 3T3 cells considered as normal fibroblasts and carcinoma MDA-MB-231, MDA-MB-435 and LNCaP cells were passaged at 2.5×10^5^ cells/25 cm^3^ flasks and extracted at different days as indicated. Day 2, 3, and 6 represent subconfluent, confluent, and post-confluent cells, respectively. B. Reduction of nucleolin mRNA in epithelial tumor cells with decreased cellular metabolism. Subconfluent MDA-MB-231 and MDA-MB-435 cells were incubated either at 37°C or 20°C for 2 and 4 hours before RNA extraction. Nucleolin mRNA expression was carried out by Q-PCR using GAPDH RNA as endogenous control mRNA. For comparative purposes, the level of nucleolin mRNA expression of the first sample in each set of experiment was considered 100% (the clear histograms). The percent decrease in nucleolin mRNA expression is given at day 2 and 3 for the 3T3 compared to day 1 in section A, and at day 4 at 20°C compared to day 4 at 37°C in section B.

The growth of normal cells is arrested due to contact inhibition with the increase in cell density, whereas tumor cells proliferate even at post-confluent densities. In view of these, we investigated the induction of nucleolin mRNA in subconfluent, confluent, and post-confluent tumor cells and compared to that observed in normal 3T3 cells, which stop proliferation once they have reached confluence (Fig. 6A). At different times after cell passage, we confirmed that the presence of nucleolin mRNA is due to an induction process as demonstrated by a marked reduction in the presence of actinomycin D (not presented). In breast and prostate carcinoma cells (MDA-MB-231, MDA-MB-435, LNCaP), nucleolin mRNA was induced constantly, and even there was a slight increase at post-confluence in the breast cancer cell lines MDA-MB-231 and MDA-MB-435 (Fig. 6A). In 3T3 cells however, nucleolin mRNA induction was reduced 40 and 60% at day 2 and 3 of cell passage that correspond to subconfluent and confluent cells, respectively (Fig. 6A). These results suggest that the induction of surface nucleolin is linked to the proliferative capacity of both normal and tumor cells. In normal cells the induction is down regulated one day after cell passage, whereas in tumor cells the induction is a continual process as long as cells are under conditions to proliferate. Indeed, when tumor cell metabolism was slowed down by incubation at 20°C for 4 hours, then there was 91 and 80% reduction of nucleolin mRNA in MDA-MB-231 and MDA-MB-435 cells respectively (Fig. 6B).

### Rapid induction of nucleolin mRNA after a cold or heat shock

**Figure 7:**
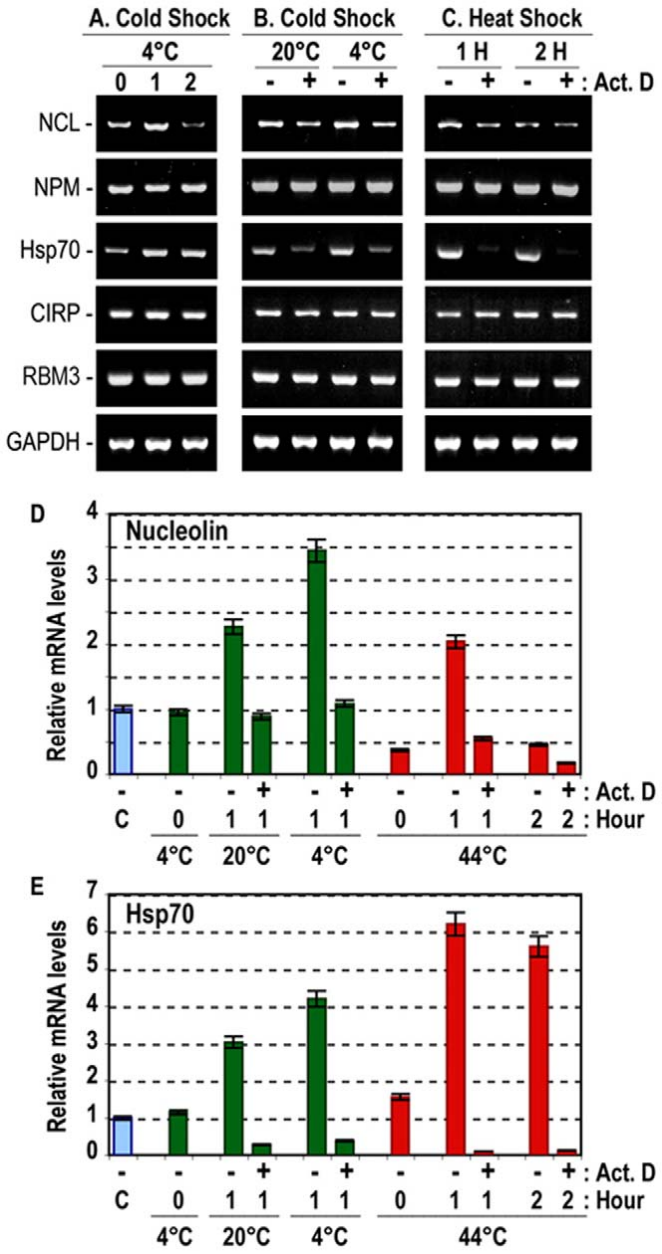
Rapid induction of nucleolin mRNA in response to cold and heat shock stress. A. Selective enhancement of nucleolin and Hsp70 mRNA expression after cold shock. Lanes 0, 1, and 2 represent RT-PCR products before, and 1 or 2 hours after cold shock at 4°C, respectively. B. Selective induction of nucleolin and Hsp70 mRNA expression after cold shock. Cells were stressed by cold shock at 20 or 4°C in the absence (lanes -) or presence (lanes +) of actinomycin D (5 µg/ml), and total RNA was prepared after 1 hour. C. Selective induction of nucleolin and Hsp70 mRNA expression after heat shock. Cells were stressed by heat shock at 44°C in the absence (lanes -) or presence (lanes +) of actinomycin D (5 µg/ml), and total RNA was prepared after 1 and 2 hours (sections1H, 2H). D/E. Relative nucleolin and Hsp70 mRNA levels after cold and heat shock. Cells in the presence or absence of actinomycin D were stressed by cold shock at 20°C or 4°C, and by heat shock at 44°C, and total RNA was extracted before (histogram C), immediately following cold or heat shock (histograms 0 hour), and 1 or 2 hours after the shock as indicated. In these experiments, MDA-MB-435 cells were processed for the cold and the heat shock treatments at two days after passage (Materials and methods). Total RNA from various samples were assayed for nucleolin (NCL), neucleophosmin (NPM), Hsp70, CIRP, RBM3 and GAPDH RNA expression by RT-PCR (in A, B, and C); the size of RT-PCR products are 835, 320, 320, 252, 396 and 983 bp, respectively. Expression of nucleolin and Hsp70 mRNA was analyzed by Q-PCR (in D and E) using GAPDH RNA as endogenous control mRNA. The ordinates give the relative mRNA expression levels compared to the control sample (histograms C), i.e. before cold or heat shock.

Analysis of nucleolin mRNA expression at low temperatures, revealed that the steady levels of nucleolin mRNA are increased consistently and concomitantly with that of the stress-induced heat shock protein Hsp70. For example, 15 min at 4°C followed by one hour incubation at room temperature (20°C) leads to enhanced expression of nucleolin and Hsp70 mRNA in MDA-MB-435 cells, whereas no apparent effect is observed on another nucleolar protein, nucleophosmin (Fig. 7A, lanes 0 and 1). Interestingly, a strong reduction of nucleolin mRNA is observed at 2 hours after the cold shock, which is in accord with the short half-life of surface nucleolin mRNA (Fig. 7A, lane 2). Two major cold-inducible RNA-binding proteins are described in human cells, CIRP (also known as A18 hnRNP) and RBM3 (for RNA binding motif protein 3) [49]. However, no apparent effect is observed on the expression of CIRP and RBM3 after 1–2 hours of the cold shock (Fig. 7A). The enhanced steady state levels of nucleolin and Hsp70 mRNA is due to a specific induction event, since they are markedly reduced with actinomycin D treatment, which has no apparent effect on the expression of nucleophosmin, CIRP, RBM3, and GAPDH (Fig. 7B).

Similarly, nucleolin mRNA is selectively induced following a heat shock stress at 44°C, an event that occurs concomitantly with a strong induction of the Hsp70 mRNA (Fig. 7C). By RT-Q-PCR analysis we further confirmed the induction of nucleolin and Hsp70 mRNA after a cold and heat shock treatment in MDA-MB-435 (Fig. 7D and 7E) and HeLa (Data not presented) cells. Overall, these studies demonstrated that cold shock at 4°C is a better inducer of nucleolin mRNA compared to heat shock, whereas heat shock is a better inducer of Hsp70 mRNA compared to cold shock. Thus, enhanced expression of nucleolin appears to be one of the immediate responses of cells to an environmental insult. Finally, it is of interest to note that 15 minutes heat but not cold shock affects directly the stability of nucleolin mRNA with more than 50% reduction (Fig. 7D, histograms 0 Hour/4°C and 0 Hour/44°C) compared to the level observed before the shock (Fig. 7D, histogram C).

### Cell surface nucleolin mediates calcium dependent internalization of ligands

HIV-1 particles and physiological ligands that bind the RGG domain of nucleolin (Midkine, pleiotrophin, lactoferrin) induce aggregation of surface nucleolin, before internalization into various cell types by an active process via lipid raft microdomains [7], [10], [17], [18], [19]. Here we used two specific ligands to illustrate calcium dependent internalization into HeLa cells through surface nucleolin: the nucleolin antagonist HB-19 pseudopeptide that binds the RGG domain, and the nucleolin specific monoclonal antibody D3 that binds the second RNA binding domain RBD2 [6], [9] (A.G.H. unpublished data). Like the physiological ligands of surface nucleolin, HB-19 and mAb D3 are internalized at 37°C but not or very little at reduced temperatures, thus further confirming that internalization via surface nucleolin occurs by an active process [6], [9]. It should be noted that internalization via surface nucleolin is a specific event, since under similar experimental conditions the basic amino acid-rich Tat peptide translocates rapidly through the plasma membrane to the nuclei and is accumulated into the nucleoli of cells as reported previously (data not presented, as in reference [50]).

**Figure 8:**
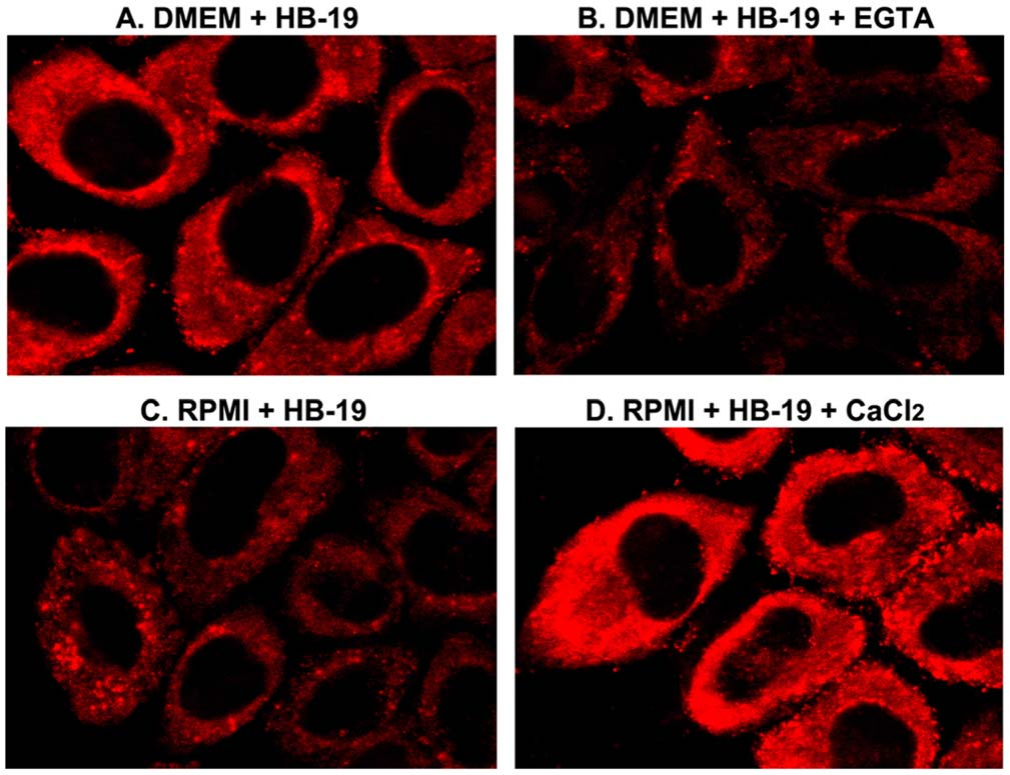
Calcium dependent internalization of HB-19 that binds the RGG domain of surface nucleolin. HeLa cells were passaged in DMEM culture medium. Two days later fresh DMEM (in A and C) or RPMI (in B and D) culture medium was added to the cell monolayer in the absence or presence of 5 mM EGTA (B) or CaCl_2_ (D) as indicated. Internalization of HB-19/Btn (5 µM) was then carried out at 37°C for 90 min before fixation with PFA-Triton and processing for confocal microscopy. Fixed cells were successively incubated with rabbit anti-biotin and goat Texas Red-conjugated anti-rabbit IgG. A scan corresponding to a cross-section toward the middle of the cell monolayer is shown.

During studies on entry of various ligands into different types of cells, we noticed that entry is more pronounced in cells cultured in DMEM compared to RPMI-1640 medium, in which the concentration of calcium is 1.8 and 0.42 mM, respectively. An example is presented for the entry of HB-19 into HeLa cells that were incubated at 37°C for 90 minutes in fresh culture medium using either DMEM or RPMI (Fig. 8A and 8C). This difference in the level of HB-19 internalization is due to the difference in the concentration of calcium, since entry is markedly reduced in DMEM-cultured cells in the presence of the extracellular calcium chelator EGTA, and inversely entry is strongly enhanced in RPMI-cultured cells supplemented with 2 mM CaCl_2_ (Fig. 8B and 8D). Similar results were observed for the internalization of midkine into DMEM-cultured cells [17] where entry is more pronounced in the presence of additional CaCl_2_, while EGTA reduces markedly the entry process (data not shown).

**Figure 9:**
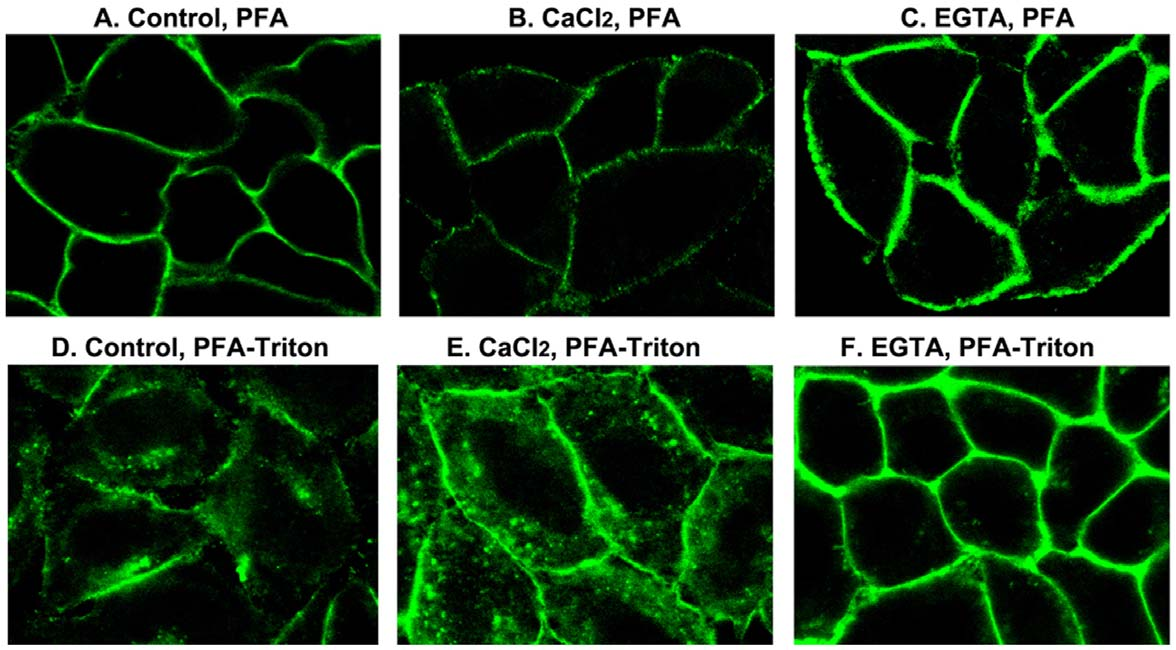
Calcium dependent internalization of surface nucleolin. DMEM-cultured HeLa cells were incubated at 37°C with mAb D3 (15 µg/ml) in the absence (A, D) or presence of additional 3 mM CaCl_2_ (B, E) or 5 mM EGTA (C, F). After 3 hours, cells were further incubated at room temperature for 90 min before PFA (7%) or PFA-Triton (7%-0.5%) fixation. The anti-nucleolin antibody mAb D3 was revealed by FITC-labeled goat anti-mouse antibodies. A scan corresponding to a cross-section toward the middle of the cell monolayer is shown.

It should be noted that in different types of cells, the cytoplasmic nucleolin signal observed by confocal immunofluorescence microscopy is very weak and is detectable only after scanning at an elevated intensity. However following ligand entry, the signal in the cytoplasm is increased and is detectable at the experimental intensity due to the internalization of the ligand-surface nucleolin complex. In order to follow the influence of calcium concentration on the translocation of surface nucleolin during the ligand entry process, we used mAb D3 that forms Triton-insoluble aggregates with surface nucleolin and is internalized at 37°C. For this purpose, cells preincubated with mAb D3 in the absence or presence of EGTA or additional CaCl_2_ were fixed with PFA to evaluate the amount of nucleolin present on the cell surface, or fixed with PFA-Triton to monitor internalization of surface nucleolin (Fig. 9). In control cells, considerable amount of nucleolin was present on the surface of control cells as well as in the cytoplasm (Fig. 9A, 9D). In the presence of the additional CaCl_2_, surface nucleolin internalization was strongly enhanced with concomitant reduction of surface nucleolin (Fig. 9B, 9E). Finally, chelating CaCl_2_ with EGTA prevented surface nucleolin internalization while at the same time it increased the surface nucleolin signal at the plasma membrane (Fig. 9C, 9F). The persistence of clustered surface nucleolin in such PFA-Triton fixed cells in the presence of EGTA indicates that chelation of CaCl_2_ does not affect translocation of the newly synthesized nucleolin towards the plasma membrane, but since internalization is blocked then nucleolin becomes accumulated in the plasma membrane probably in triton insoluble microdomains. Taken together, our data demonstrate that calcium plays an important role in the internalization process of ligands via surface nucleolin.

## Discussion

By monitoring the expression of nucleolin mRNA and by measuring separately the level of surface, cytoplasmic and nuclear nucleolin protein, we demonstrate here that nucleolin mRNA is constantly induced in tumor cells to synthesize nucleolin that is rapidly translocated to the cell surface. The estimated half-life of nucleolin mRNA and cytoplasmic/surface nucleolin is about 90 and 45 minutes, respectively, whereas the half-life of nuclear nucleolin could be more than 24 hours as reported before [47]. Surface/cytoplasmic nucleolin therefore is differentially expressed compared to its nuclear counterpart. Consistent with this, the reduced levels of nucleolin mRNA in response to inhibition of RNA transcription is accompanied by marked reduction of surface and cytoplasmic but not nuclear nucleolin. Others have reported that surface nucleolin is the product of nuclear nucleolin translocation to the cell surface by comparing enhanced levels of surface nucleolin or nucleolin mRNA with the level of nucleolin protein in total cell lysates, i.e., mixture of nuclear, cytoplasmic and surface nucleolin [14], [47]. The proportion of the steady state levels of surface nucleolin in different types of cell is 5–10% of nucleolin found in total cell extracts [6], [46]. Consequently, the measurement of total cellular nucleolin protein cannot be used as an indication of the changes in surface nucleolin, since they can lead to inaccurate interpretation of the relationship between nuclear and cell-surface presence of nucleolin. The constant induction of surface nucleolin under various growth and stress conditions described herein, indicate that future investigations on nuclear nucleolin should take into consideration the potential contribution of surface nucleolin, in order to provide a more comprehensive analysis of experimental observations.

Since the first report of surface expression of nucleolin in hepatocarcinoma cells [51], emerging evidences suggest that cell-surface nucleolin is implicated in cell proliferation, tumor cell growth and angiogenesis. The enhanced expression of surface nucleolin is observed *in vitro* and *in vivo* in lymphoid organs containing activated lymphocytes, on the surface of tumor and endothelial cells, or in angiogenic endothelial cells within the tumor vasculature [6], [13], [31], [52]. The expression of nucleolin mRNA has been systematically detectable in peripheral blood lymphocytes of healthy individuals, and *in vitro* stimulation of cells leads to an enhanced expression of nucleolin mRNA and surface nucleolin [31], [53], [54]. Here we demonstrate that enhanced expression of surface nucleolin in tumor cells is due to its induction that is highly associated to the state of cell proliferation. Indeed, nucleolin mRNA induction is reduced markedly in normal fibroblasts that reach confluence, while it occurs continuously even in post-confluent epithelial tumor cells consistent with their capacity to proliferate without contact inhibition. When cell metabolism is slowed down at reduced temperature in epithelial tumor cells, then nucleolin mRNA induction is reduced markedly but it is resumed rapidly once cells are returned back to 37°C. In endothelial cells (HUVECs), we show that the enhanced expression of nucleolin mRNA is associated with the induction of surface nucleolin in response to activation of cells, such as in response to VEGF. Accordingly, expression of nucleolin on the surface of HUVECs is markedly reduced in cultures incubated with actinomycin D for few hours. Previously by laser scanning confocal immunofluorescence microscopy we have demonstrated that the expression of surface nucleolin is enhanced strongly in activated cells, while it is reduced markedly in growth-arrested cells and in cells cultured with actinomycin D or cycloheximide. Interestingly, such modifications in the expression of surface nucleolin occur in the absence of any apparent effect on the level and nuclear localization of nuclear nucleolin [6], thus illustrating once again that expression of surface nucleolin occurs continuously compared to its nuclear counterpart.

Treatment of cells with the nucleolin antagonist HB-19 pseudopeptide results in a selective reduction of surface/cytoplasmic nucleolin without any apparent effect on the level or nucleolar localization of nuclear nucleolin [13]. In such HB-19 treated cells, nucleolin mRNA is continuously induced but it is not translated. The molecular mechanism of such a specific translational block on nucleolin mRNA in the absence of nucleolin protein remains to be investigated. However, it is tempting to speculate that nucleolin mRNA might require the nucleolin protein for its translation as it is the case for metalloproteinase-9 (MMP-9) and *bcl2* oncogene mRNA [4], [55], [56]. Indeed, nucleolin present in the cytoplasm binds 3′-untranslated region in the mRNA of MMP-9 and Bcl-2, a process that is necessary for the stability and translational efficiency of these mRNAs [4], [5]. Nucleolin-binding to MMP-9 mRNA increases the production of the enzyme that by degrading extracellular matrix components promotes tumor metastasis, whereas in B-cell chronic lymphocytic leukemia cells the increased levels of cytoplasmic nucleolin is directly related to overexpression of the *bcl2* oncogene that blocks apoptosis.

Surface nucleolin serves as a low affinity receptor for HIV-1 and various growth factors, such as midkine, pleiotrophin, and lactoferrin [7], [10], [17], [18], [19]. Binding of these ligands results in clustering of cell-surface nucleolin in lipid raft membrane microdomains before endocytosis of the ligand-nucleolin complex by an active process. Accordingly, surface nucleolin could shuttle ligands between the cell surface and the nucleus thus act as a mediator for the extracellular regulation of nuclear events [18], [20], [21]. Here we show that ligand internalization is increased in the presence of elevated calcium concentrations in the culture medium, whereas chelation of calcium inhibits internalization, thus indicating that extracellular calcium plays a key role in the mechanism of surface nucleolin mediated active internalization of nucleolin-ligands. Besides its well-documented role in the internalization of specific ligands, we recently reported that ligand binding to surface nucleolin could also induce calcium entry into cells, a process inhibited by chelating calcium from the culture medium [15]. It might therefore be possible that ligand triggered calcium entry is necessary for the rearrangement of actin cytoskeleton that affect the actions of contractile proteins, as well as being a cofactor for the activation of a number of intracellular signaling pathways [57]. As Nucleolin is shown to bind Ca^2+^ [58], [59], and is involved in epidermal Ca^2+^ homeostasis [60], it remains to be shown whether binding of calcium to surface nucleolin is a necessary step for ligand internalization via surface nucleolin. In view of this, its implication in different metabolic events, its capacity to bind various pathogens and diverse range of ligands including low density lipoproteins, it is plausible to suggest that surface nucleolin functions as scavenger receptors [3], [51], [61], [62]. Interestingly, surface nucleolin expression and ligand internalization via surface nucleolin has often been associated with the expression of the low-density lipoprotein receptor-related protein-1, a multifunctional scavenger receptor that mediates the uptake of various ligands from the pericellular environment [7], [12], [20], [63].

In addition to its association with tumor cell proliferation, here we show that cold or heat shock stress affect the expression of nucleolin mRNA. This is due to a specific induction but not stabilization of nucleolin mRNA, since it is prevented by actinomycin D in the absence of an apparent effect on the expression of the mRNA of another nucleolar protein, nucleophosmin. Interestingly, enhanced expression of nucleolin mRNA after a cold or heat shock occurs concomitantly with the enhanced expression of Hsp70 mRNA. On the other hand, no apparent effect is observed on the expression of the two major cold-shock induced proteins CIRP and RBM3, which like nucleolin contain RNA binding motifs and a C-terminal glycine rich domain, and are up-regulated in human tumors [3], [49]. The simultaneous induction of nucleolin and Hsp70 mRNA has also been shown to occur during early stage of liver regeneration [64], [65], thus suggesting that enhanced expression of nucleolin and Hsp70 is not just fortuitous to cold and heat shock treatment of cells *in vitro*, but it may occur *in vivo* in response to physiologically relevant stress and proliferation events.

Previously, nuclear nucleolin and nucleophosmin were reported to be part of the cellular response to genotoxic stress, and several stress-responsive transcripts that are potentially regulated by nucleolin have been identified [66]. In response to DNA damage conditions or heat shock, a significant fraction of nucleolar nucleolin was shown to form a complex with the replication protein A, an essential factor in many DNA processing reactions [67], [68]. Intracellular nucleolin has also been reported to be a crucial downstream effector of Hsp70 in the protection of cardiomyocytes against oxidative stress-induced apoptosis [69]. Hsp70 family of stress proteins are ubiquitous and highly conserved proteins whose expression is induced in response to a wide variety of physiological and environmental insults, such as heat shock, oxidative stress, anticancer drugs [70], and cold shock as shown here. Hsp70 has a dual function depending on its intracellular or extracellular location [70]. Intracellular Hsp70 with other heat shock proteins play an essential role as molecular chaperones by assisting the correct folding of nascent and stress-accumulated misfolded proteins, whereas extracellular Hsp70 is thought to be internalized via scavenger receptors to promote cell survival by stabilizing the membranes of lysosomes [71], [72], [73]. In view of this latter and the concomitant induction of Hsp70 and surface nucleolin under stress conditions, the potential implication of surface nucleolin in the antiapoptotic effect of Hsp70 remains to be determined.

Several reports have now provided evidence that surface nucleolin is a promising target for cancer therapy [13], [37], [40], [74], [75]. Chemotherapy by targeting surface nucleolin could be less toxic compared to conventional cancer drugs, since nucleolin is continuously and abundantly expressed in tumor compared to normal cells, thus making tumor cells the preferential targets of inhibitors of surface nucleolin. Another parameter that could contribute to the lack of toxicity is the capacity of an inhibitor to block the functioning of surface nucleolin without affecting nuclear nucleolin, which controls many aspects of gene expression [1], [3]. In this respect, HB-19 treatment is not toxic *in vitro* and *in vivo* [13], [37], [52]. After specifically binding to surface nucleolin, HB-19 enters cells and accumulates in the cytoplasm but it does not cross the nuclear membrane. Consequently, the effect of HB-19 is exerted differentially via the cell surface expressed nucleolin without affecting nuclear nucleolin.

## Materials and Methods

### Cells and culture medium

Human breast (MDA-MB-231, MDA-MB-435 [13]), prostate (LNCaP, from American Type Culture Collection, ATCC, Rockville, MD), and cervical (HeLa [76]) epithelial cancer cells were grown in DMEM-glutamax medium (Gibco Invitrogen, Cergy-Pontoise, France) containing GlutaMAX™, 4.5 g/l glucose and supplemented with 10% heat-inactivated (56°C for 30 min) fetal bovine serum (FBS; Hyclone, Thermo Fisher Scientific, Inc). G401 nephroblastoma cells (ATCC) were cultured in McCoy's (Gibco) containing 2 mM L-glutamine and supplemented with 10% heat-inactivated FBS. Jurkat (acute T cell leukemia [77]), HuT 78 (cutaneous T lymphocytes [78]) and CEM (acute lymphoblastic leukemia, CEM/clone 13 provided by Luc Montagnier [78]) were cultured in RPMI-1640 medium (Gibco) containing GlutaMAX™, and supplemented with 10% heat-inactivated FBS. Cells were kept at logarithmic growth phase and used at a cell density of 2-5×10^5^/ml. 3T3 cells (mouse embryonic fibroblast, ATCC) were cultured in DMEM medium without pyruvate containing 4.5 g/l glucose and 10% bovine calf serum (BCS, Gibco). Human umbilical vein endothelial cells (HUVECs) were provided by José Courty (from Clonetics; Biowhittaker, Emerain-ville, France) and cultured between passages 2 and 5 in EBM-2 Bullekit medium (Biowhittaker) supplemented with 2% bovine calf serum (BCS). All cultures were grown at 37°C in a humidified atmosphere under 5% CO_2_.

### Peptide constructs

The HB-19 pseudopeptide 5[Kψ(CH_2_N)PR-TASP, for [Lysψ(CH_2_N)Pro-Arg]-template-assembled synthetic peptide, binds specifically surface nucleolin and block its function. The template in HB-19, presents pentavalently the tripeptide 5[Kψ(CH_2_N)PR where (CH_2_N) represents a reduced peptide bond between lysine and proline residues. The synthesis of HB-19 and biotinylated HB-19 (HB-19/Btn; this is used for the recovery of surface nucleolin) were synthesized by Jean Paul Briand as described previously using solid phase peptide methodology [16], [79]. All peptides were obtained at a high purity (95%), and their integrity was controlled by matrix-associated laser desorption ionization-time-of-flight analysis. The synthetic fluorescein isothiocyanate (FITC)-coupled Tat (amino acid residues 48–60) with the sequence of FITC-Ahx-YGRKKRRQRRRPPQS-OH was provided by Jean Paul Briand, IBMC, Strasbourg.

### Preparation of cytoplasmic and nuclear extracts

Cells washed in phosphate-buffered saline (PBS) were lysed in buffer E (20 mM Tris-HCl, pH 7.6, 150 mM NaCl, 5 mM MgCl_2_, 5 mM β-mercaptoethanol, protease inhibitor cocktail (Sigma) and 0.5% Triton X-100) and the nuclei were pelleted by centrifugation (1000 *g* for 5 min). For the preparation of nuclear extracts, the nuclear pellet was disrupted in buffer I (20 mM Tris-HCl, pH 7.6, 50 mM KCl, 400 mM NaCl, 1 mM EDTA, 5 mM β-mercaptoethanol, protease inhibitor cocktail, 1% Triton X-100, and 20% glycerol). The nucleus-free supernatants and the nuclear extracts were then centrifuged at 12,000 *g* for 10 min, and the supernatants were stored at −20°C. Aliquots of crude cell extracts were diluted in 2 fold concentrated electrophoresis sample buffer containing SDS, heated and analyzed by SDS-polyacrylamide gel electrophoresis (PAGE) [9], [10].

### Analysis of the cell-surface-expressed nucleolin

Two days after seeding, subconfluent cells (about 5×10^6^ cells/75 cm^2^ flask) were incubated (45 min, 20°C) with 5 µM of HB-19/Btn. After washing extensively in PBS containing 1 mM EDTA (PBS-EDTA), nucleus-free cell extracts were prepared in lysis buffer E. The complex formed between cell-surface expressed nucleolin and HB-19/Btn was isolated by purification of the extracts using NeutrAvidin agarose (100 µl; Pierce Biotechnology) in PBS-EDTA. After 3 hours at 6°C, the avidin-agarose samples were washed extensively with PBS-EDTA. The purified surface nucleolin was eluted in the electrophoresis sample buffer containing SDS and analyzed by 10% SDS–polyacrylamide gel electrophoresis (PAGE). The presence of nucleolin was then revealed by immunoblotting using mAb D3 against nucleolin as described before [10], [16]. The presence of actin was monitored with mAb anti-actin A-4700 from Sigma. To estimate the half-life of the nucleolin protein, the intensity of nucleolin protein bands (100 kDa) was quantified by using the NIH image software. To monitor the profile of proteins in crude cells extracts, gels were stained with Brilliant Blue G-Colloidal Concentrate from Sigma.

### Laser scanning confocal immunofluorescence microscopy

Cells were plated 24 hours before the experiment in eight-well glass slides (Lab-Tek Brand; Nalge Nunc International, Naperville, IL). Cells were fixed with either paraformaldehyde (3.7% PFA, 10 min) for membrane staining to monitor the clustered surface nucleolin or PFA/Triton X-100 (10 min 3.7% PFA, 3 washing with PBS, and permeabilization using 0.5% Triton solution) for staining intracellular HB-19/Btn or nucleolar nucleolin [6]. For labeling of surface nucleolin, cells were incubated with mAb D3 for 45 min at room temperature before PFA fixation and processing for confocal immunofluorescence microscopy. Incubation at room temperature prevents intracellular translocation of antibody-nucleolin complex and allows antibody-dependent clustering of surface nucleolin [6]. The secondary antibodies were the following: FITC-conjugated goat anti-mouse IgG (Sigma), Rabbit anti-biotin concentrate (IgG fraction; Enzo Diagnostics, Inc., New York), Texas Red dye-conjugated goat anti-rabbit IgG (Jackson ImmunoResearch Laboratories).

### Cold and heat shock treatment of MDA-MB-435 and HeLa cells

For the cold shock treatment, subconfluent cells in 25 cm^3^ flasks were cooled by transfer to a refrigerator at 4°C for 10–15 min, during which time the temperature of the culture medium becomes reduced gradually to 24, 16 and 12°C after 5, 10, and 15 min, respectively. Cells were then left at room temperature (20–22°C) for 1–6 hours as indicated in individual experiments before extraction of total RNA. For the heat shock treatment, cells were heated for 15 min by immersion of the flasks in a water bath at 44°C. Cells were then left at room temperature for the indicated times before RNA extraction. In order to monitor the net induction of nucleolin mRNA after cold and heat shock, the recovery of cells was carried out at room temperature, since at 37°C there is constant induction of nucleolin mRNA.

### Nucleolin mRNA expression monitored by reverse transcription-polymerase chain reaction (RT-PCR)

Adherent cells (NIH 3T3, MDA-MB-231, MDA-MB-435, G401, HeLa, LNCaP) were passaged in Dulbecco's Modified Eagle Medium (DMEM) culture medium containing 10% FCS in 6-well plates. After 2 days, subconfluent cells were stimulated by replacing the culture medium by DMEM containing 10% FCS. Such stimulated cells in the absence or presence of actinomycin D (5 µg/ml), cycloheximide (50 µg/ml), or tunicamycin (10 µg/ml) were cultured for 3–5 hours before extraction for total RNA using RNeasy Mini Kit (Qiagen) according to the manufacturer's instructions. Leukemia cells (HuT 78, Jurkat, CEM) passaged in RPMI culture medium containing 10% FCS were suspended at 5×10^5^ cells/ml in the absence or presence of inhibitors for 4–5 h before preparation of RNA extracts. RT was carried out with oligo(dT) and 2–4 µg of total RNA using Superscript II Reverse Transcriptase (Invitrogen). PCR was performed in a RoboCycler 96 (Stratagene, La Jolla, CA, USA) with specific primers for human nucleolin (5′-TTGAATTCATCATGGTGAAGCTCGCGAAGGC-3′ and 5′-TAGGGCCCAGGCTCTTCCTCCTC-3′), glyceraldehyde-3-phosphate dehydrogenase (GAPDH) (5′-TGAAGG-TCGGAGTCAACGGATTTGGT-3′ and 5′-CATGTGGGCCATGAGGTCCA-CCAC-3′), nucleophosmin (5′- TGGTTCTCTTCCCAAAGTGG-3′ and 5′-TAAAACCAAGCAAAGGGTGG-3′), cold shock protein CIRP (5′-GGGTCGTTGTGGTGCGCTGT-3′ and 5′- TGGCATCCTTAGCGTCGTCA-3′), cold shock protein RBM3 (5′- CGCAGCCCCGTCCCTGTTTT-3′ and 5′-GTAGCTGCGACCACGCCCAT-3′), and heat shock protein Hsp70 (5′-AAGGTGGAGATCATCGCCAA-3′ and 5′-GCGATCTCCTTCATCTTGGT-3′). PCR amplification conditions were: 95°C for 2 min, 30 cycles of 95°C for 30 sec, 60°C for 30 sec and 72°C for 45 sec, and 72°C for 5 min. The intensity of nucleolin bands was quantified by using the NIH image software.

### Real Time quantitative PCR (Q-PCR)

Q-PCR amplification was performed in a 7900HT fast real-time PCR System (Applied Biosystems, Foster City, CA) using 384 well plates. For each reaction, each individual sample was run in triplicate wells with 5 µl of ABSOLUTE™ QPCR SYBR Absolute QPCR Green Rox Mix (Thermo Scientific), 0.2 µl of 10 µM forward and reverse primer (giving a final concentration of 200 nM), 1 µl of cDNA at 40 or 80-fold dilution, and 3.6 µl of water. Non-template controls were run in triplicate for each primer master mix. Q-PCR amplification parameters were the following: an initial denaturation at 95°C for 10 min, followed by 40 cycles with denaturation at 95°C for 5 s, and the annealing/elongation at 60°C for 30 s, followed a dissociation stage at 95°C 15 s, 60°C 15 s and 95°C 15 s. The expression of nucleolin (NCL) mRNA was investigated with primers specific for Human (Hu) and murine (Mu) nucleolin in human and mouse cells respectively. For endogenous control RNA expression, we used oligonucleotide primers specific for 28S ribosomal RNA (rRNA) and for human and murine GAPDH. The primer sequences were designed using the primer blast site at NCBI. The following primers were used: Hu NCL (5′-AGGAGGAGGAAGAAGAGGAG-3′ and 5′- ACAAAGAGATTGAAAGCCGTAG-3′; product size 148 bp); Hu GAPDH (5′-GCACCGTCAAGGCTGAGAA-3′ and 5′-AGGGATCTCGCTCCTGGAA-3′; product size 75 bp); Mu NCL (5′-AAGCAGCACCTGGAAAACG-3′ and 5′-TCTGAGCCTTCTACTTTCTGTTTCTTG-3′; product size 85 bp); Mu GAPDH (5′-GAACATCATCCCTGCATCCA-3′ and 5′-CCAGTGAGCTTCCCGTTCA-3′; product size 78 bp); rRNA 28S (5′- GTAAAACTAACCTGTCTCACG-3′ and 5′- AAGCAGGAGGTGTCAGAAA-3′; product size 199 bp); Hu Hsp70 (5′- AACCGCACCACCCCCAGCTA-3′ and 5′- TCGCCGAACTTGCGGCCAAT-3′; product size 137 bp). The relative mRNA expression was calculated using the comparative C(T) method also referred to as the 2 (-DeltaDeltaC(T)) method [80]. 28S ribosomal RNA or GAPDH RNA were used as endogenous controls for RNA expression. Q-PCR results are expressed in relative levels or as percentage of their respective controls (means ± SD).

## References

[1] Srivastava M, Pollard HB (1999). Molecular dissection of nucleolin's role in growth and cell proliferation: new insights.. FASEB J.

[2] Ginisty H, Sicard H, Roger B, Bouvet P (1999). Structure and functions of nucleolin.. J Cell Science.

[3] Storck S, Shukla M, Dimitrov S, Bouvet P (2007). Functions of the histone chaperone nucleolin in diseases.. Subcell Biochem.

[4] Fahling M, Steege A, Perlewitz A, Nafz B, Mrowka R (2005). Role of nucleolin in posttranscriptional control of MMP-9 expression.. Biochim Biophys Acta.

[5] Otake Y, Soundararajan S, Sengupta TK, Kio EA, Smith JC (2007). Overexpression of nucleolin in chronic lymphocytic leukemia cells induces stabilization of bcl2 mRNA.. Blood.

[6] Hovanessian AG, Puvion-Dutilleul F, Nisole S, Svab J, Perret E (2000). The cell-surface-expressed nucleolin is associated with the actin cytoskeleton.. Exp Cell Res.

[7] Hovanessian AG (2006). Midkine is a cytokine that inhibits HIV infection by binding to the cell surface expressed nucleolin.. Cell Res.

[8] Callebaut C, Blanco J, Benkirane N, Krust B, Jacotot E (1998). Identification of V3 loop-binding proteins as potential receptors implicated in the binding of HIV particles to CD4^+^ cells.. J Biol Chem.

[9] Nisole S, Said EA, Mische C, Prevost MC, Krust B (2002). The anti-HIV pentameric pseudopeptide HB-19 binds the C-terminal end of nucleolin and prevents anchorage of virus particles in the plasma membrane of target cells.. J Biol Chem.

[10] Nisole S, Krust B, Hovanessian AG (2002). Anchorage of HIV on permissive cells leads to co-aggregation of viral particles with surface nucleolin at membrane raft microdomains.. Exp Cell Res.

[11] Sinclair JF, O'Brien AD (2002). Cell surface-localized nucleolin is a eukaryotic receptor for the adhesin intimin-gamma of enterohemorrhagic Escherichia coli O157:H7.. J Biol Chem.

[12] Barel M, Hovanessian AG, Meibom K, Briand JP, Dupuis M (2008). A novel receptor - ligand pathway for entry of Francisella tularensis in monocyte-like THP-1 cells: interaction between surface nucleolin and bacterial elongation factor Tu.. BMC Microbiol.

[13] Destouches D, El Khoury D, Hamma-Kourbali Y, Krust B, Albanese P (2008). Suppression of tumor growth and angiogenesis by a specific antagonist of the cell-surface expressed nucleolin.. PLoS ONE.

[14] Huang Y, Shi H, Z H, Song X, Yuan S (2006). The angiogenesis function of nucleolin is mediated by vascular endothelial growth factor and nonmuscle myosin.. Blood.

[15] Losfeld ME, El Khoury D, Mariot P, Carpentier M, Krust B (2009). The cell surface expressed nucleolin is a glycoprotein that triggers calcium entry into mammalian cells.. Exp Cell Res.

[16] Nisole S, Krust B, Callebaut C, Guichard G, Muller S (1999). The anti-HIV pseudopeptide HB-19 forms a complex with the cell-surface expressed nucleolin independent of heparan sulfate proteoglycans.. J Biol Chem.

[17] Said AE, Krust B, Nisole S, Briand JP, Hovanessian AG (2002). The anti-HIV cytokine midkine binds the cell-surface-expressed nucleolin as a low affinity receptor.. J Biol Chem.

[18] Legrand D, Vigie K, Said EA, Elass E, Masson M (2004). Surface nucleolin participates in both the binding and endocytosis of lactoferrin in target cells.. Eur J Biochem.

[19] Said EA, Courty J, Svab J, Delbé J, Krust B (2005). Pleiotrophin inhibits HIV infection by binding the cell surface expressed nucleolin.. FEBS J.

[20] Shibata Y, Muramatsu T, Hirai M, Inui M, Kimura T (2002). Nuclear targeting by the growth factor midkine.. Mol Cell Biol.

[21] Stepanova V, Lebedeva T, Kuo A, Yarovoi S, Tkachuk S (2008). Nuclear translocation of urokinase-type plasminogen activator/.. Blood.

[22] Kadomatsu K, Muramatsu T (2004). Midkine and pleiotrophin in neural development and cancer.. Cancer Lett.

[23] Perez-Pinera P, Berenson JR, Deuel TF (2008). Pleiotrophin, a multifunctional angiogenic factor: mechanisms and pathways in normal and pathological angiogenesis.. Curr Opin Hematol.

[24] Dumler I, Stepanova V, Jerke U, Mayboroda OA, Vogel F (1999). Urokinase-induced mitogenesis is mediated by casein kinase 2 and nucleolin.. Current Biology.

[25] Kleinman HK, Weeks BS, Cannon FB, Sweeney TM, Sephel GC (1991). Identification of a 110-kDa nonintegrin cell surface laminin-binding protein which recognizes an A chain neurite-promoting peptide.. Arch Biochem Biophys.

[26] Turck N, Lefebvre O, Gross I, Gendry P, Kedinger M (2006). Effect of laminin-1 on intestinal cell differentiation involves inhibition of nuclear nucleolin.. J Cell Physiol.

[27] Larrucea S, Gonzalez-Rubio C, Cambronero R, Ballou B, Bonay P (1998). Cellular adhesion mediated by factor J, a complement inhibitor. Evidence for nucleolin involvement.. J Biol Chem.

[28] Harms G, Kraft R, Grelle G, Volz B, Dernedde J (2001). Identification of nucleolin as a new L-selectin ligand.. Biochem J.

[29] Reyes-Reyes EM, Akiyama SK (2008). Cell-surface nucleolin is a signal transducing P-selectin binding protein for human colon carcinoma cells.. Exp Cell Res.

[30] Tate A, Isotani S, Bradley MJ, Sikes RA, Davis R (2006). Met-independent hepatocyte growth factor-mediated regulation of cell adhesion in human prostate cancer cells.. BMC Cancer.

[31] Christian S, Pilch J, Akerman ME, Porkka K, Laakkonen P (2003). Nucleolin expressed at the cell surface is a marker of endothelial cells in angiogenic blood vessels.. J Cell Biol.

[32] Shi H, Huang Y, Zhou H, Song X, Yuan S (2007). Nucleolin is a receptor that mediates antiangiogenic and antitumor activity of endostatin.. Blood.

[33] Grinstein E, Wernet P (2007). Cellular signaling in normal and cancerous stem cells.. Cell Signal.

[34] Alete DE, Weeks ME, Hovanessian AG, Hawadle M, Stoker AW (2006). Cell surface nucleolin on developing muscle is a potential ligand for the axonal receptor protein tyrosine phosphatase-sigma.. FEBS J.

[35] Di Segni A, Farin K, Pinkas-Kramarski R (2008). Identification of nucleolin as new ErbB receptors- interacting protein.. PLoS ONE.

[36] Inder KL, Lau C, Loo D, Chaudhary N, Goodall A (2009). Nucleophosmin and nucleolin regulate K-Ras plasma membrane interactions and MAPK signal transduction.. J Biol Chem.

[37] El Khoury D, Destouches D, Lengagne R, Krust B, Hamma-Kourbali Y (2010). Targeting Surface Nucleolin with a Multivalent Pseudopeptide Delays Development of Spontaneous Melanoma in RET Transgenic Mice.. BMC Cancer.

[38] Kato M, Takahashi M, Akhand AA, Liu W, Dai Y (1998). Transgenic mouse model for skin malignant melanoma.. Oncogene.

[39] Xu X, Hamhouyia F, Thomas SD, Burke TJ, Girvan AC (2001). Inhibition of DNA replication and induction of S phase cell cycle arrest by G-rich oligonucleotides.. J Biol Chem.

[40] Bates PJ, Laber DA, Miller DM, Thomas SD, Trent JO (2009). Discovery and development of the G-rich oligonucleotide AS1411 as a novel treatment for cancer.. Exp Mol Pathol.

[41] Soundararajan S, Wang L, Sridharan V, Chen W, Courtenay-Luck N (2009). Plasma membrane nucleolin is a receptor for the anticancer aptamer AS1411 in MV4-11 leukemia cells.. Mol Pharmacol.

[42] Girvan AC, Teng Y, Casson LK, Thomas SD, Jüliger S (2006). AGR0100 inhibits activation of nuclear factor-kappaB (NF-kappaB) by forming a complex with NF-kappaB essential modulator (NEMO) and nucleolin.. Mol Cancer Ther.

[43] Teng Y, Girvan AC, Casson LK, Pierce WM, Qian M (2007). AS1411 alters localization of a complex containing protein arginine methyltransferase 5 and nucleolin.. Cancer Res.

[44] Soundararajan S, Chen W, Spicer EK, Courtenay-Luck N, Fernandes DJ (2008). The nucleolin targeting aptamer AS1411 destabilizes Bcl-2 messenger RNA in human breast cancer cells.. Cancer Res.

[45] Callebaut C, Jacotot E, Krust B, Guichar G, Blanco J (1997). Pseudopeptides TASP inhibitors of HIV infection block viral entry by binding to a 95 kDa cell surface protein.. J Biol Chem.

[46] Carpentier M, Morelle W, Coddeville B, Pons A, Masson M (2005). Nucleolin undergoes partial N- and O-glycosylations in the extranuclear cell compartment.. Biochemistry.

[47] Kim SK, Srivastava M (2003). Stability of nucleolin protein as the basis for the differential expression of nucleolin mRNA and protein during serum starvation.. DNA and Cell Biol.

[48] Gattoni-Celli S, Buckner CL, Lazarchick J, Stuart RK, Fernandes DJ (2009). Overexpression of nucleolin in engrafted acute myelogenous leukemia cells.. Am J Hematol.

[49] LLeonart ME (2010). A new generation of proto-oncogenes: Cold-inducible RNA binding proteins.. Biochim Biophys Acta.

[50] Vivès E, Brodin, Lebleu B (1997). A truncated HIV-1 Tat protein basic domain rapidly translocates through the plasma membrane and accumulates in the cell nucleus.. J Biol Chem.

[51] Semenkovich CF, Ostlund REJ, Olson MO, Yang JW (1990). A protein partially expressed on the surface of HepG2 cells that binds lipoproteins specifically is nucleolin.. Biochemistry.

[52] Krust B, Vienet R, Cardona A, Rougeot C, Jacotot E (2001). The anti-HIV pentameric pseudopeptide HB-19 is preferentially taken up in vivo by lymphoid organs where it forms a complex with nucleolin.. Proc Natl Acad Sci USA.

[53] Westmark CJ, Malter JS (2001). Up-regulation of nucleolin mRNA and protein in peripheral blood mononuclear cells by extracellular-regulated kinase.. J Biol Chem.

[54] Callebaut C, Nisole S, Briand JP, Krust B, Hovanessian AG (2001). Inhibition of HIV infection by the cytokine midkine.. Virology.

[55] Otake Y, Tapas K, Sengupta S, Bandyopadhyay S, Spicer EK (2005). Retinoic-induced apoptosis in HL-60 cells is associated with Nucleolin down-regulation and destabilization of Bcl-2 mRNA.. Molecular Pharmacology.

[56] Otake Y, Soundararajan S, Sengupta TK, Kio EA, Smith JC (2010). Overexpression of nucleolin in chronic lymphocytic leukemia cells induces stabilization of bcl2 mRNA.. Neoplasia.

[57] Meledez AJ, Tay HK (2008). Phagocytosis: a repertoire of receptors and Ca(2+) as a key second messenger.. Biosci Rep.

[58] Sorokina EA, Kleinman JG (1999). Cloning and preliminary characterization of a calcium-binding protein closely related to nucleolin on the apical surface of inner medullary collecting duct cells.. J Biol Chem.

[59] Gilchrist JS, Abrenica B, DiMario PJ, Czubryt MP, Pierce GN (2002). Nucleolin is a calcium-binding protein.. J Cell Biochem.

[60] Hwang J, Kalinin A, Hwang M, Anderson DE, Kim MJ (2007). Role of Scarf and its binding target proteins in epidermal calcium homeostasis.. J Biol Chem.

[61] Pluddemann A, Neyen C, Gordon S (2007). Macrophage scavenger receptor and host-derived ligands.. Methods.

[62] Hirano K, Miki Y, Hirai Y, Sato R, Itoh T (2005). A multifunctional shuttling protein nucleolin is a macrophage receptor for apoptotic cells.. J Biol Chem.

[63] Lillis AP, Van Duyn LB, Murphy-Ullrich JE, Strickland DK (2008). LDL receptor-related protein 1: unique tissue-specific functions revealed by selective gene knockout studies.. Physiol Rev.

[64] Ohmori H, Murakami T, Furutani A, Higashi K, Hirano H (1990). Simultaneous activation of heat shock protein (hsp 70) and nucleolin genes during in vivo and in vitro prereplicative stages of rat hepatocytes.. Exp Cell Res.

[65] Konishi T, Karasaki Y, Nomoto M, Ohmori H, Shibata K (1995). Induction of heat shock protein 70 and nucleolin and their intracellular distribution during early stage of liver regeneration.. J Biochem.

[66] Yang C, Maiguel DA, Carrier F (2002). Identification of nucleolin and nucleophosmin as genotoxic stress-responsive RNA-binding proteins.. Nucleic Acids Res.

[67] Iliakis G, Krieg T, Guan J, Wang Y, Leeper D (2004). Evidence for an S-phase checkpoint regulating DNA replication after heat shock: a review.. Int J Hyperthermia.

[68] Kim K, Dimitrova DD, Carta KM, Saxena A, Daras M (2005). Novel checkpoint response to genotoxic stress mediated by nucleolin-replication protein A complex formation.. Mol Cell Biol.

[69] Jiang B, Zhang B, Liang P, Song J, Deng H (2010). Nucleolin/C23 mediates the antiapoptotic effect of heat shock protein 70 during oxidative stress.. FEBS J.

[70] Schmitt E, Gehrmann M, Brunet M, Multhoff G, Garrido C (2007). Intracellular and extracellular functions of heat shock proteins: repercussions in cancer therapy.. J leukoc Biol.

[71] Thériault JR, Adachi H, Calderwood SK (2006). Role of scavenger receptors in the binding and internalization of heat shock protein 70.. J Immunol.

[72] Nylandsted J, Gyrd-Hansen M, Danielewicz A, Fehrenbacher N, Lademann Y (2004). Heat shock protein 70 promotes cell survival by inhibiting lysosomal membrane permeabilization.. J Exp Med.

[73] Kirkegaard T, Roth AG, Petersen NH, Mahalka AK, Olsen OD (2010). Hsp70 stabilizes lysosomes and reverts Niemann-Pick disease-associated lysosomal pathology.. Nature.

[74] Folkman J (2007). Endostatin finds a new partner: nucleolin.. Blood.

[75] Fogal V, Sugahara KN, Ruoslahti E, Christian S (2009). Cell surface nucleolin antagonist causes endothelial cell apoptosis and normalization of tumor vasculature.. Angiogenesis.

[76] Nisole S, Krust B, Dam E, Blanco A, Seddiki N (2000). The HB-19 pseudopeptide 5[Kψ(CH_2_N)PR]-TASP inhibits attachment of T-lymphocyte- and macrophage-tropic HIV to permissive cells.. AIDS Res Hum Retroviruses.

[77] Jacotot E, Callebaut C, Blanco J, Rivière Y, Krust B (1996). HIV envelope glycoprotein-induced cell killing by apoptosis is enhanced with increased expression of CD26 in CD4+ T cells.. Virology.

[78] Rey MA, Krust B, Laurent AG, Guetard D, Montagnier L (1989). Characterization of an HIV-2 related virus with a smaller size extracellular envelope glycoprotein.. Virology.

[79] Callebaut C, Jacotot E, Guichard G, Krust B, Rey-Cuille MA (1996). Inhibition of HIV infection by pseudopeptides blocking viral envelope glycoprotein-mediated membrane fusion and cell death.. Virology.

[80] Schmittgen TD, Livak KJ (2008). Analyzing real-time PCR data by the comparative C(T) method.. Nat Protoc.

